# Higher Prevalence of p16-Positive OPSCC Among Males in Northern Nevada: A Regional Epidemiological Study

**DOI:** 10.3390/jcm15135060

**Published:** 2026-06-29

**Authors:** Samantha Chang, Steven T. Cho, Nicholas Bazett, Sydney Denney, Stefan Harspter, Nadya Vinsdata, Dylan Wrye, Katharine Thomas

**Affiliations:** 1 Reno School of Medicine, University of Nevada, Reno, NV 89557, USA; stevencho@med.unr.edu (S.T.C.); nbazett@med.unr.edu (N.B.); sydneyalthoff@med.unr.edu (S.D.); sharpster@med.unr.edu (S.H.); nvinsdata@med.unr.edu (N.V.); dwrye@med.unr.edu (D.W.); 2 Pennington Cancer Institute, Reno, NV 89502, USA; katharine.thomas@renown.org

**Keywords:** HPV, p16, OPSCC, sex disparity, HPV vaccination, Nevada

## Abstract

**Background/Objectives**: Human papillomavirus (HPV) is a well-established risk factor for oropharyngeal squamous cell carcinoma (OPSCC), with p16 expression serving as a reliable surrogate marker for HPV involvement and an important prognostic indicator. While data exist on the demographic and clinical features of p16-positive OPSCCs on a national scale, limited data exist for Nevada. **Methods**: This retrospective study evaluated the relationship between p16 positivity and OPSCC presentation in Northern Nevada, with specific attention to associations between p16 status, sex, socioeconomic factors, smoking history, and presenting symptoms. We reviewed medical records of 296 patients diagnosed with OPSCC with concomitant HPV testing at the Renown Regional Medical Center from 2014–2024. Variables of interest included sex, age at diagnosis, smoking pack-years, Nevada census-based income quintile, county of residence, and initial symptoms such as dysphagia and sore throat. Multivariate logistic regression was performed to assess associations between these variables and p16 status. **Conclusions**: We found that sex was the only variable significantly associated with p16 positivity: females had significantly lower odds of being p16-positive compared to males (OR: 0.36, 95% CI: 0.20–0.65, *p* = 0.001). This finding of sex-based disparity in HPV-related OPSCC is consistent with national trends. This study is limited by its regional focus and lack of data on other social determinants of health. Nonetheless, the results underscore the importance of gender-informed prevention strategies and highlight the need for further research into the biological and behavioral contributors to this disparity.

## 1. Introduction

Human Papillomavirus (HPV) is a leading cause of oropharyngeal squamous cell carcinoma (OPSCC). HPV-positive OPSCC has reached up to 72.6% prevalence, but researchers note differing regional trends [1,2]. This highlights the importance of understanding local HPV epidemiology. As HPV causes cancer through altered p16 tumor suppressor regulation, p16 testing has become a surrogate marker and the standard prognostic indicator for HPV-related OPSCC. Consequently, the results of p16 testing can direct treatment choices and intensity, with direct impacts on patient outcomes and quality of life [3].

Several studies have identified varying demographic patterns associated with p16 positivity. Research by Scott-Wittenborn et al. and C. Ferreira et al. shows rising p16-positive OPSCC prevalence over time and that p16 positivity is associated with younger age, higher education levels, and non-smoking status [4,5]. Chaturvedi et al. observed increasing incidence and survival among HPV-positive patients, a trend confirmed in older adults by Windon et al., who noted that the prognostic value of p16 testing is consistent through different age ranges [6,7]. SEER data showed that males and individuals of higher socioeconomic status were more likely to have p16-positive OPSCC. On a global scale, notable geographic variability in HPV-related cancer prevalence exists, highlighting inequities in access, diagnosis, and prevention [8,9]. These demographic differences highlight the importance of p16 testing for informing care but also suggest that HPV trends differ based on various socioeconomic and geographic factors. These findings justify the need for localized research to understand HPV-mediated OPSCC in distinct populations and regions.

Despite growing national attention, Northern Nevada remains underrepresented in HPV-related OPSCC research. By evaluating regional demographics and clinical presentation in p16-positive OPSCC cases, this study addresses a gap in the literature and may help inform targeted public health strategies in the Northern Nevada region. It also provides the rationale for the research hypothesis: that p16-positive OPSCC is more prevalent among males in Northern Nevada, independent of socioeconomic status, smoking history, or presenting symptoms.

Globally, there has been a dramatic increase in the rate of HPV-positive OPSCC, with reports of a 225% increase from 1988 to 2004 alone. HPV now accounts for up to 20–25% of OPSCC cases [1,2]. However, this rate varies drastically depending on region. Meta-analyses have revealed HPV^+^ OPSCC rates ranging from 10% to 85%, typically with higher rates in more developed countries. In the United States, HPV accounts for about 70% of newly diagnosed OPSCC [3,4,5]. Additionally, men see drastically increased rates of HPV-related OPSCC versus females, likely secondary to overall higher rates of HPV infection in men [6,7,8]. Despite rising prevalence of HPV-related cancers and a WHO recommendation of HPV vaccination since 2008, only 125 countries (64%) have incorporated HPV vaccination guidelines into national policy. Of these countries, only 47 (37.6%) have gender-neutral guidelines [10]. These further underscores the need for further epidemiological studies that highlight the growing prevalence of HPV.

## 2. Materials and Methods

The study protocol was reviewed by the Institutional Review Board (IRB) of the University of Nevada, Reno, who granted a waiver of informed consent for a retrospective study of deidentified data. A list of all OPSCC patients diagnosed or treated at Renown Regional Medical Center (RRMC) from 2000–2024 was obtained through RRMC’s Pennington Cancer Institute. Patients without p16 testing or with cancers other than in the oropharynx were excluded from the analysis. An Electronic Medical Record (EMR) was then used to collect information from the remaining patients. Collected information included clinical characteristics (tumor location, tumor size, node status, and metastasis (TNM), p16 status, and staging. Patient staging was verified via the latest guidelines from the American Joint Committee on Cancer (AJCC) 8th edition. Demographic data including sex, age at diagnosis, smoking pack-years, county of residence, and presenting symptoms (dysphagia and sore throat) were collected for the remaining cohort. Patients missing any of the above data were also excluded from the study.

Self-reported zip codes were used to determine patients’ county of residence. Socioeconomic status was estimated using their county of residence combined with mean income data from the US Census. Five-year estimates of county-level Nevada income data, reflecting average values from 2018 to 2022, were obtained from data.census.gov using the most recent 2023 release. County-level mean household income was extracted from Table S1901 “Income in the Past 12-Months (in 2023 Inflation-Adjusted Dollars)”, while income quintile distributions for Nevada counties were obtained from Table B19081 “Mean Household Income of Quintiles”. Patients were then sorted into income quintiles using their reported county of residence, with quintiles 1 and 5 representing the lowest and highest socioeconomic quintiles, respectively. Income data were unavailable for Nye and Humboldt counties, and patients from those counties were excluded from economic variable analyses. Logistic regression models assessed associations between p16 positivity and the selected variables. All analyses were performed using the Stata 18.5 Basic Edition software (StataCorp., College Station, TX, USA). Results were reported as odds ratios (ORs) with 95% confidence intervals (CIs) and corresponding *p*-values, with statistical significance defined as *p* < 0.05. Associations between p16 status and these categorical variables were evaluated using chi-squared (χ^2^) tests. Statistical significance was defined as *p* < 0.05.

## 3. Results

Logistic regression was performed to evaluate the association between p16 expression and multiple variables (Table 1). Sex was significantly associated with p16 positivity, with females showing lower odds than males (OR: 0.36, 95% CI: 0.20–0.65, *p* = 0.001). Other variables, including mean income quintile (OR: 1.13, 95% CI: 0.75–1.72, *p* = 0.562), county (OR: 1.10, 95% CI: 0.62–1.95, *p* = 0.742), dysphagia (OR: 0.76, 95% CI: 0.45–1.27, *p* = 0.297), and sore throat (OR: 1.13, 95% CI: 0.68–1.89, *p* = 0.633), were not significantly associated with p16 expression. Additionally, neither age at diagnosis (OR: 1.00, 95% CI: 0.98–1.03, *p* = 0.805) nor smoking pack-years (OR: 1.00, 95% CI: 0.99–1.01, *p* = 0.683) showed statistically significant associations with p16 status (Table 2).

## 4. Discussion

In this retrospective cohort study, we found that male sex was significantly associated with p16-positive OPSCC in Northern Nevada (*p* < 0.001; OR: 0.36). This was independent of socioeconomic status, smoking history, or presenting symptoms. These findings are consistent with national data showing a significantly growing burden of HPV-related OPSCC among men and suggest that sex-specific biological or behavioral factors may contribute to this disparity [6,7,8]. Notably, other variables traditionally considered risk factors for OPSCC, such as income level and geographic area of residence, were not significantly associated with p16 status in our population. This suggests the predictive utility of traditional social determinants in HPV-mediated OPSCC may have regional variability or may need revisions based on the changing social environment.

Overall, our results align with existing literature that highlights male sex as a key predictor of HPV-mediated OPSCC. However, unlike the findings of Chaturvedi et al. and Jordan et al., our analysis did not show a significant relationship between p16 status and socioeconomic quintile. This discrepancy may reflect regional differences in access to care, HPV vaccination rates, or population demographics [4,9]. Additionally, we did not find a correlation between the presence of sore throat or dysphagia and p16 positivity, suggesting limited clinical utility for symptom-based screening in this population. However, a broader analysis of cancer symptoms will be necessary before this can be concluded.

Our data are also consistent with global trends suggesting that men and higher income countries have higher prevalence of HPV^+^ OPSCC. This growing disparity highlights the need for further action into HPV prevention in these cohorts. Despite WHO recommendation for HPV vaccination since 2008, only 47% (*n* = 125) of countries worldwide have implemented HPV vaccination guidelines into national policy. Of these countries, only 37% (*n* = 47) have gender-neutral vaccination guidelines [11,12].

The observed sex-based disparity in HPV-related OPSCC underscores the need for more targeted prevention efforts. Despite the availability of effective HPV vaccines, uptake among males has historically lagged behind female vaccine uptake, contributing to higher rates of HPV-mediated malignancies in men [13]. HPV awareness and willingness to receive the HPV vaccine were significantly lower among male college students compared to females, with 85.3% of males vs. 93.2% of females having heard of HPV and 55.9% of males vs. 80.4% of females willing to receive the HPV vaccine [14]. This imbalance may be due to past vaccine marketing focused primarily on preventing cervical cancer in women, leading to lower awareness and perceived relevance among men [15]. Multiple studies have identified key barriers to HPV vaccination in boys and men, including lack of knowledge, vaccine hesitancy, cost, and misconceptions about the vaccine promoting promiscuity [16]. Our findings suggest further need for HPV vaccination campaigns and increased awareness among men.

This study has several limitations. As a single-center retrospective analysis, our findings may not be fully generalizable to other regions or healthcare systems. The absence of HPV DNA testing is another limitation, although p16 remains a validated surrogate marker in clinical practice [17]. Furthermore, we lacked data on several potentially relevant social determinants of health, such as education level, occupational exposure, and marital status. Additionally, we used county-level income as a crude proxy for individual socioeconomic status (SES). While county-level income is widely variable and may not accurately represent the full demographics of SES, more specific data were not available for immediate use. Further analysis into correlations between SES, HPV status, and OPSCC treatment outcomes may further delineate healthcare disparities.

HPV vaccination status is another important consideration for OPSCC that was notably missing from our study. Since its first release in 2006, the HPV vaccine has emerged as the gold standard preventative for HPV-related cancers. Despite initially aimed at preventing cervical cancers, a 2024 study found that HPV vaccination is associated with decreased rates of all HPV-related cancers [18]. Analyzing how social determinants of health affect HPV vaccine uptake will be especially important for future studies with rising rates of vaccine hesitancy and as the first cohort of vaccine recipients reaches adulthood. These variables may offer additional insight into the mechanisms underlying sex-based HPV transmission disparities.

Future research should investigate the biological, behavioral, and systemic factors driving the overrepresentation of men in HPV-related OPSCC. Regional studies that incorporate vaccination records, sexual health history, and viral genotyping could help clarify these trends [19]. Our findings support the implementation of gender-informed prevention and screening strategies in Northern Nevada and reinforce the importance of continued local surveillance to guide public health efforts [20].

## 5. Conclusions

This study demonstrates that the epidemiology of HPV-mediated OPSCC in Northern Nevada reflects national trends, with a significantly higher prevalence of p16 positivity among men. Conventional factors such as smoking history, income level, and presenting symptoms did not correlate with HPV-related disease, highlighting the limitations of these variables in risk assessment. These findings underscore the need for targeted and gender-informed prevention strategies, particularly through expanded HPV vaccination and education efforts aimed at male populations. Given the continued rise of HPV-related OPSCC, especially in underserved regions, region-specific research and tailored public health interventions are essential to reduce disparities and improve outcomes.

While the study’s regional scope and retrospective design limit causal inference and generalizability beyond Northern Nevada, the findings reinforce the utility of p16 as a reliable prognostic and epidemiologic marker. Moving forward, precision public health strategies that account for sex differences and local population needs should guide the next phase of HPV-related cancer prevention and control.

## Figures and Tables

**Table 1:** Income data were unavailable for Nye and Humboldt counties; therefore, 9 patients (3.0%) were excluded from income analyses (*N* = 287). Data are presented as *n* (%) unless otherwise indicated.

Variable	Category	*n* (%)
p16 Status	Positive	94 (31.8)
	Negative	202 (68.2)
Sex	Male	236 (79.7)
	Female	60 (20.3)
County	Washoe	213 (72.0)
	Other	83 (28.0)
Race	White	281 (94.9)
	Non-White	15 (5.1%)
Income Quintile (*N* = 287) †	1st (USD 23,700)	2 (0.7)
	2nd (USD 42,400)	24 (8.4)
	3rd (USD 53,100)	170 (59.2)
	4th (USD 65,500)	91 (31.7)
	5th (USD 101,500)	0 (0.0)
Sore Throat	Present	134 (45.4)
	Absent	161 (54.6)
Dysphagia	Present	158 (53.6)
	Absent	137 (46.4)
Pack-Years	Mean ± SD	19.9 ± 20.5
Age at Diagnosis (years)	Mean ± SD	63.2 ± 10.3

† *N* = 287 here as nine participants were excluded from analysis as income quartile data for their county of residence was not available.

**Table 2:** Male sex was significantly associated with p16-positive OPSCC. No other demographic or clinical variables were significantly associated with p16 positivity. Odds ratios (ORs), 95% confidence intervals (CIs), and *p*-values were obtained using univariate logistic regression. Income data were unavailable for Nye and Humboldt counties; therefore, 9 patients (3.0%) were excluded from income analyses (*N* = 287). Statistical significance was defined as *p* < 0.05.

Variable	Comparison	OR	95% CI	*p*-Value
Sex	Female vs. Male	0.36	0.20–0.65	0.001
County	Other Counties vs. Washoe County	1.10	0.62–1.96	0.742
Dysphagia	Present vs. Absent	0.76	0.45–1.27	0.297
Sore Throat	Present vs. Absent	1.13	0.68–1.89	0.633
Income Quintile	Per-quintile increase	1.13	0.75–1.71	0.560

## Data Availability

Patient data compounded using internal RRMC databases and are not publicly available. Interested parties are encouraged to reach out regarding data sharing.

## Abbreviations

The following abbreviations are used in this manuscript:

HPV	Human Papillomavirus
EMR	Electronic Medical Record
SEER	Surveillance, Epidemiology, and End Results Database
OPSCC	Oropharyngeal Squamous Cell Carcinoma
RRMC	Renown Regional Medical Center
SES	Socioeconomic Status
